# Pre-roling: an operational framework for facilitators and simulating participants (SPs) to prepare for both acting and educating safely

**DOI:** 10.1186/s41077-026-00419-w

**Published:** 2026-02-23

**Authors:** Casper Danholt Iuul, Peter Dieckmann, Birgitte Bruun

**Affiliations:** 1 Department of Communication, Center for Tracking & Society (CTS), University of Copenhagen, Karen Blixens Plads 8, Copenhagen, 2300 Denmark; 2 Department of Engineering Technology and Didactics, Strategy & Leadership Development, Danish Technical University (DTU), Lautrupvang 15, Ballerup, Denmark; 3 Copenhagen Academy for Medical Education and Simulation (CAMES), Borgmester Ib Juuls Vej 1, opg. 1, etage 25, Herlev, DK-2730 Denmark

**Keywords:** Simulation, Medical education, Fieldwork, Ethnography, Action theory

## Abstract

Sometimes scenario-based simulations do not go as expected because facilitator and simulating participant (SP) are not sufficiently aligned. This innovation paper presents a pre-roling framework that offers an operational, theoretically informed tool for facilitators and SPs to prepare systematically for scenario-based simulations in health professions education. The framework development was based on four months of ethnographic fieldwork at the Copenhagen Academy for Medical Education and Simulation (CAMES) and informed by action theory.

The pre-roling framework guides facilitators and SPs through five analytically distinct aspects of simulation performance — meaning (educational purpose), narrative (intended course of events), style (tone, tempo), character (the portrayed person), and health professions education (clinical picture, learning environment, task distribution, and care for the SP). Facilitators hold primary responsibility for meaning, narrative and style, while SPs hold primary responsibility for the character, with explicit collaboration across all domains.

The framework and its accompanying pocket card and worksheet were iteratively refined in dialogue with SPs, course leaders and scenario designers. It is intended as a flexible, context-sensitive preparation tool that can be used symmetrically by SPs and facilitators, from novice to experienced, and in diverse simulation settings. It fills a previously unoccupied space between general guidelines and the moment of action by systematizing preparation without prescribing content, thereby supporting clearer alignment, more deliberate improvisation, and explicit attention to SP safety. Using the framework can reduce dissonance between SPs and facilitators, enhance the coherence of dramaturgical and educational intentions, improve learners’ experiential learning opportunities, and help prevent distress among SPs.

**Supplementary Information:**

The online version contains supplementary material available at 10.1186/s41077-026-00419-w.

“…the facilitators are just all different. Even if I did it exactly as one facilitator would have it, the next facilitator might want it in a whole other way”.

Simulating participant in scenario-based simulation training.

## Introduction

This innovation paper offers a framework for ‘pre-roling’^1^, which is the systematic preparation by simulating participants (SPs^2^) and facilitators for scenario-based simulations in health professional education (HPE). The literature already offers excellent advice on training SPs for role portrayal [1–3] and, more broadly, on how to engage SPs in simulations [4, 5]. Dedicated associations offer detailed guidelines and standards for engaging SPs in healthcare simulations [6–8]. In our experience, however, it remains a challenge in practice for SPs and facilitators to stay on the same track once scenarios are rolling. A part of this challenge stems from the inevitable degree of improvisation that is inherent and necessary in scenario-based simulations to keep them on track [9]. Another part of this challenge stems from the need for SPs and facilitators to understand each other’s responsibilities, particularly if SPs or facilitators are new to their task, if they have not worked together before, or if the scenario is new. When facilitators and SPs are not aligned the quality of simulations may become uneven, which in turn can reduce the learning potential for learners^3^. In addition, SPs may immerse themselves so much in their character, take so much responsibility for learners’ experience, and try to do such a good job for the facilitator, that they risk compromising their own physical and mental well-being [10, 11].

In response to these challenges, we have developed an operational framework for SPs and facilitators to pre-role in a way that systematically and explicitly prepares for both the dramaturgical aspects of the simulation, HPE as the overall purpose of the simulation, and the collaboration between SP and facilitator. The pre-roling framework can be applied whether SPs embody a character in the same space as learners or give voice to the character of a mannikin from behind a screen.

The pre-roling framework was developed by CDI based on ethnographic fieldwork among SPs working at the Copenhagen Academy for Medical Education and Simulation (CAMES) in Denmark. Fieldwork materials were filtered through theory about action by professional actors [12]. Theatre theories have inspired simulation in HPE in many ways, often in relation to building and enacting convincing characters [3, 13–15]. In this innovation paper, however, we are inspired by theory about the division of labour between instructor and professional actor, whom in simulation would be facilitator and SP.

Although the pre-roling framework is built on insights into SPs’ tasks the tool is designed to be equally relevant for both SPs and facilitators. The pre-roling framework can be applied by all SPs and facilitators engaging in scenario-based simulations, but it is perhaps particularly useful in structuring preparations for novice SPs and facilitators. It can be useful in settings where new scenarios are often developed, where new ‘pairs’ of facilitators and SPs are often established, where facilitators and SPs engage in many different scenarios, or as a checklist for simulation scenario designers. In addition, it can be useful when working with professional actors, who are not accustomed to applying their skills for educational purposes.

The pre-roling framework adds to existing guidance in several ways. As a hands-on tool it fits into a previously uninhabited space between general guidelines for simulation and the moment of action. Just as importantly, the pre-roling framework can be applied ’symmetrically’ by SPs and facilitators. In this way the pre-roling framework enables an increasing, but not always operationalized, acknowledgement of SPs as important partners for facilitators. In addition, the framework explicitly reminds both the facilitator and the SP to pay attention to potential risks to the SP’s physical and mental well-being while at work. Attention to SPs’ wellbeing at work is not new at all [10, 11, 16, 17], but the pre-roling framework focuses on the particular kinds of risk to SPs’ well-being that may stem from lack of alignment with facilitators.

## The framework development process

Scenario-based simulation is applied in many HPE settings each with their own combination of institutional set-up, learning goals, simulation and debriefing methods, types of learners, and programmes for recruiting and training facilitators and SPs. Local practices vary. The pre-roling framework was developed in response to needs in a particular setting that we briefly present before we describe the ethnographic research project and the action theory that inspired the design of the framework.

As a unit in the Capital Region of Denmark, CAMES runs simulation-based courses for students and healthcare professionals coming from different healthcare and healthcare education organisations and at all stages of their careers. Several courses include scenario-based simulations for team training and many health professionals return to CAMES several times during their education programmes. Learning goals in these simulations always include knowledge of medical procedures, treatment or care and elements of social and cognitive competencies [18]. Scenario scripts are written with these learning goals in mind, and several forms of fidelity are built around them [19]. Realism and (to some degree) standardization are seen as means towards learning, not as goals in themselves. This means that there is some leeway for adapting standard scenarios to the specific learning needs of learners and, at times, the preferences of individual facilitators.

Scenario-based simulations at CAMES are run by a corps of approximately 100 clinicians who have all participated in a three-day facilitator course and course-specific further training, but who vary in both their simulation and clinical experience. A group of around 40 medical students work as operators (the person controlling the simulation computer and other technical aspects of the scenario, and who might also give voice to characters during scenarios, e.g., patients, or staff working in the simulated organization) behind a one-way windowpane, as patients in the beds of the simulation centre, or in other relevant settings outside the simulation centre [20]. SPs are not typecasted in terms of age or history but learn from courses and from their peers how to embody various patients with different symptoms. Some facilitators invite SPs to observe learners to provide feedback to them in the subsequent debriefing. As medical students graduate, approximately 12–14 new students are recruited each year from as early as their first year in medical school. Many students stay in their job at CAMES until they graduate.

CDI conducted four months of ethnographic fieldwork at CAMES (late August to late November), with a focus on SPs’ training and day-to-day work across both onboarding and routine course delivery. During the study period, the SP workforce comprised 38 medical-student SPs (working as operators and as in-role SPs), collaborating with the facilitator corps. Fieldwork combined participant observation during scheduled course days and SP shifts with informal “hanging out” in the spaces where SP work and coordination occurred, including the morning meeting point, operator rooms, simulation rooms, briefing/debriefing areas, and informal breaks (coffee and lunch). Apart from about 100 h of observation, empirical materials included informal conversations with 31 of the 38 SPs (7 men, 24 women), as well as conversations with facilitators and permanent CAMES staff, supplemented by nine in-depth semi-structured interviews (1–2 h) with SPs (eight women and one man). The fieldwork was timed to coincide with the recruitment and onboarding of 11 newly hired SPs; six of the interviewees were novices, while the remaining three had approximately one year of experience, allowing comparison across experience levels. Based on observations of both novice and experienced SPs’ daily work supplemented with interviews, CDI quickly saw how the supposedly “same” scenarios (according to the script) developed quite differently, for example, because learners made different choices or because facilitators differed in their emphases on various elements in the scenarios. Simulations usually worked well in spite of this variation, but CDI also saw how much effort SPs put into making the scenarios convincing and engaging; how the division of responsibilities between SPs and facilitators was sometimes unclear; and how SPs continuously tried to “read” their facilitator’s preferences with regard to various aspects of the scenarios.

To understand the dynamics in the relation between SP and facilitator CDI consulted Kirsten Hastrup’s anthropological exploration of Shakespeare actors’ work [12] which is informed by the theatre actor Simon Callow’s theory of acting and of the clear division of labour between director and actor [21]. This division of labour is predicated upon four distinct aspects of any performance, i.e. meaning, narrative, style and character. Based on Hastrup and Callow’s insights, CDI developed a framework, and a graphic representation of it, for analytically separating the same four aspects, which SP and facilitator could benefit from addressing together before running a scenario-based simulation. During the development of the framework the researcher group regularly discussed it with course developers, scenario designers and SPs at CAMES to make it more applicable. From these discussions ‘’health professional education” was added as the pivot of the framework. CDI presented a complete version of the pre-roling framework for SPs and course developers, who welcomed its clarity and immediate appeal as an organizing tool for the training of new SPs, new facilitators, and course developers, as well as for the design and conduct of simulation scenarios. The version presented in this paper is the result of continuous refinement built upon feedback from facilitators, SPs, and workshop participants in Denmark and at international simulation and HPE conferences, as well as reviewers of this paper.

## Theoretical foundations: acting and anthropological action theory

Looking at the division of labour between the SP and the facilitator during simulations, CDI found a helpful way of thinking about performance in the writings of the British actor Simon Callow, who proposes dividing theatre performance into four distinct aspects, i.e. meaning, narrative, style and character. Meaning captures the deeper themes or messages that the narrative is meant to convey. Narrative refers to the ordered sequence of events and actions that give the performance its plot. Style denotes the rhythmic framing of the action - the tone, tempo and physicality that shape how the story feels. Character is the actor’s embodied enactment of a specific persona-psychology, habits, voice and gestures. Callow argues that all four aspects interact with each other, and that they are evenly important to create a successful play [21].

The anthropologist, Kirsten Hastrup, builds on these concepts in her theory of action, which proposes how actions create social worlds [12]. By exploring how Shakespeare actors perform and by extending her insights about their actions to everyday situations, her theory of action is based on a blurring of the distinction between “real world” and performance – just like simulations that only “work” if they are perceived as real and not real at the same time [14, 22, 23] and where SPs often draw from their personal experience when enacting a role [24].

In Hastrup’s study of how actors act, she builds on an important point made by Callow about the division of labour between actor and director. Callow and Hastrup argues that ‘character’ is mainly the responsibility of the actor, whereas narrative, meaning and style are the responsibility of the director. This division of responsibilities rests on differences in the nature of their relationship with the performance. The director is responsible for the global view of the situation, for seeing the bigger picture and for steering the performance in the direction of the desired outcome. To use a metaphor: The director looks at a detailed map and is in charge of planning the itinerary for the actor [12]. The actor, on the contrary, is immersed in the world of the performance and is therefore not able to keep a complete overview of the situation. The actor is responsible for attuning to every twist and turn within the play and to act in truthful agreement with the character. However, professional actors also need to know which way to go to reach the right destination and are therefore dependent on the instructions of the director. The director has to make the narrative, meaning and style of the play evident to the actor, but leave the character to the actor in order to let the actor unfold the character within the frames of the director’s instructions (ibid.:200).

## Applying action theory to scenario-based simulations

Examining fieldwork data with this theory of the division of labour in mind - directors being responsible for narrative, meaning and style, whereas actors are responsible for character - made it clear to us why so many SPs, particularly novices, spent so much energy trying to ‘read’ their facilitators once the scenario was running. It appeared that facilitators were not always sufficiently explicit as directors of the scenario in terms of narrative, meaning or style before the scenario took place, so SPs resorted to looking for guidance from the facilitator as the scenario unfolded. Indeed, we saw how SPs work from moment to moment to attune not only to their character, but also to learners and to the facilitator [25].

Furthermore, the analytical separation of the four aspects of a performance allowed us to name and specify some dimensions in scenario scripts that were otherwise often muted and that could be addressed more explicitly when preparing simulation scenarios.

Translating *director* into *facilitator* and *actor* into *SP*, we have transferred Hastrup’s theory of action to the performance of scenario-based simulations. Accordingly, we propose to look at scenario-based simulations as a matter of meaning, narrative, style, and character to enable a more explicit dialogue between facilitator and SP about expectations to the scenario. To accommodate health professional education as the main purpose of healthcare simulation we propose this as a fifth aspect with its own significance in terms of preparation and practicalities.

**Fig. 1:**
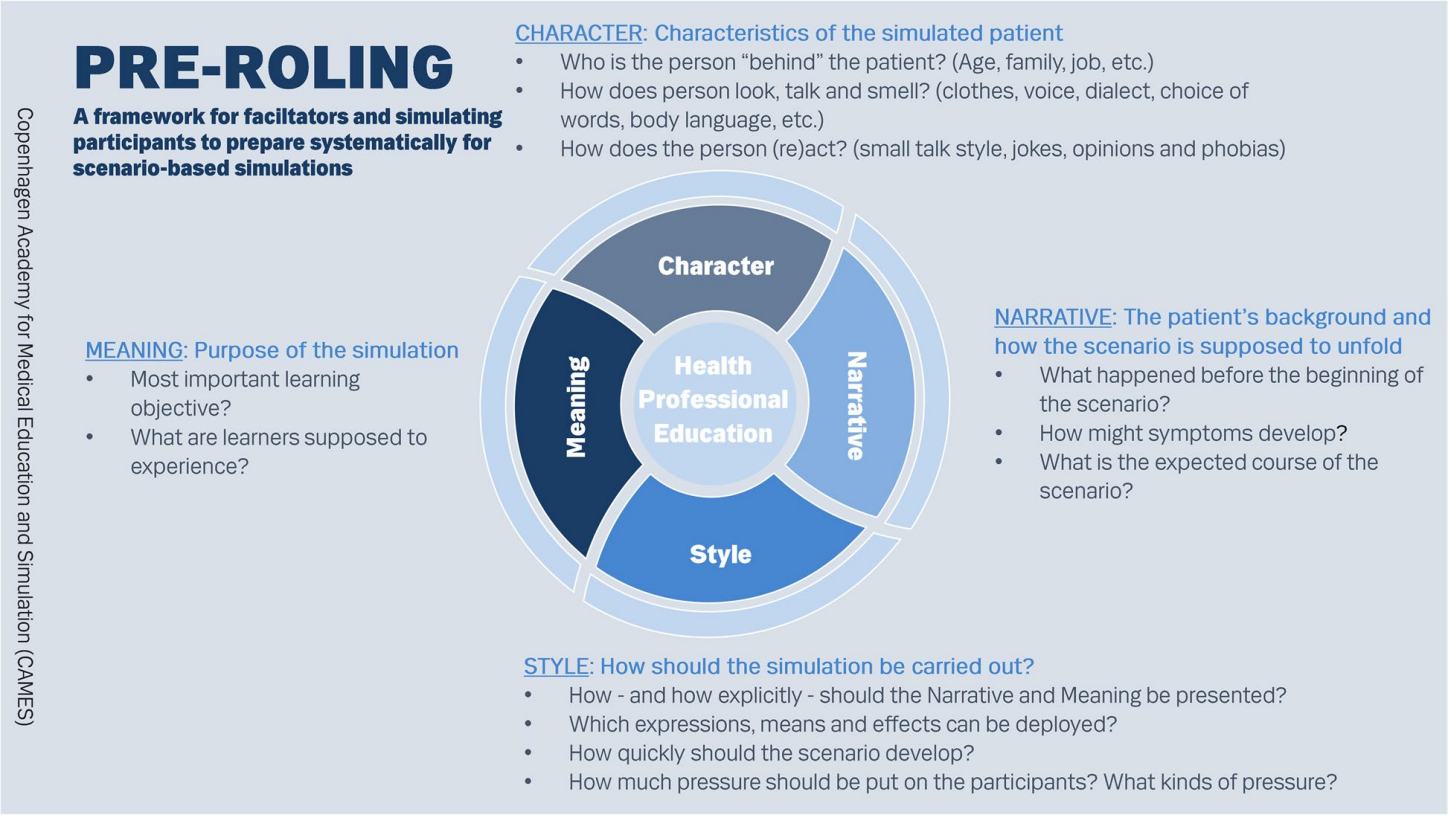
Pre-roling pocket card - front

**Fig. 2:**
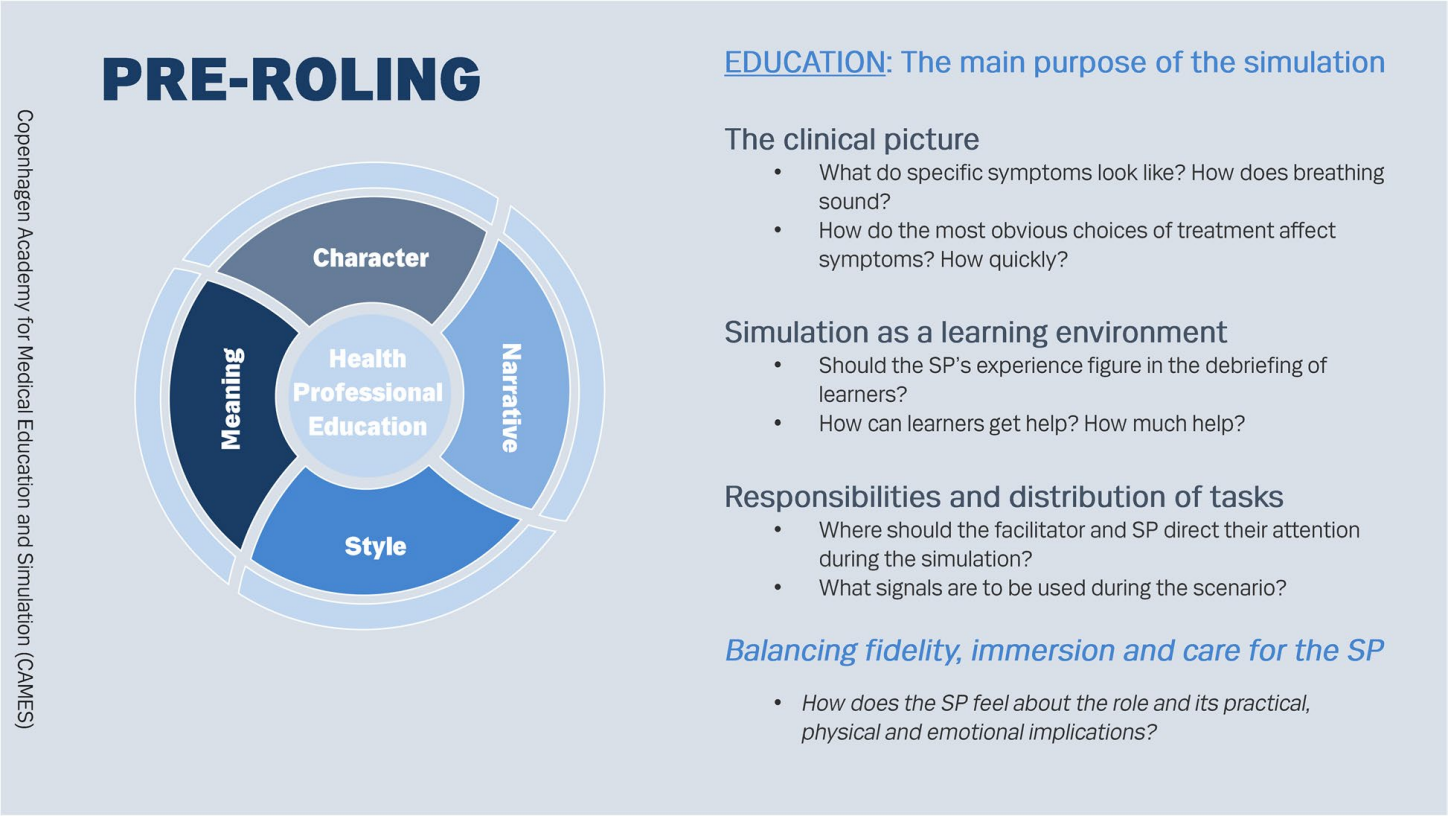
Pre-roling pocket card – back

We have condensed the ideas in the pre-roling framework into a graphic representation of a wheel with four spokes around a pivot as presented on the pocket card, Figs. 1 and 2 (additional file 1). We have also found it useful to transform the pocket card into a table for facilitators and/or SPs to fill in and share for each scenario as an aid to systematically making assumptions more explicit (additional file 2).

The pre-roling framework describes five different aspects of the simulation performance. Since the most common figure for SPs in CAMES to enact is a patient, the pocket card refers to this figure, but the basic ideas are applicable for other figures as well.

## The pre-roling framework and its five aspects

### Meaning: purpose of the simulation

Meaning is about the overall purpose of the simulation. If the purpose of the simulation is not clear for the SP, it is difficult to act in accordance with it and to enable that this meaning stands out from all other impressions.

Most SP’s have experienced a scenario, where the main purpose of the simulation was unclear, which made it difficult for them to improvise during the scenario. One SP described a transport scenario, where she was in a bed being run along a hospital floor. The team had not brought the oxygen along for transportation and only discovered this when the patient needed oxygen as per the scenario script. The learners started to pretend that they were giving the patient oxygen. This places the SP in a difficult position. It was unclear to her if the meaning of simulation was to teach the learners to bring all the vital equipment for transportation, or if the meaning was to see how they would work together when unforeseen complications occurred during transportation. She decided to play along with the imagined oxygen mask. However, when they arrived back with the facilitator, he made a point out of the fact that they had forgotten to pack the oxygen before leaving the room. Since the SP played along instead of challenging the learners by passing out, they did not experience the failure of not checking the bed for essential equipment before leaving.

For this reason, the first question in the meaning aspect is as follows:

- What is the most important learning objective?

The expectation that SPs know learning objectives may seem too obvious to mention, but some scenarios have more than one learning objective that can be weighed differently; some facilitators may (wrongly) assume that SPs are already informed about which learning goal should be in focus for the upcoming scenario; and some SPs run many scenarios in one day and become unsure. For the SP to feel confident and authentic, the SP needs to know what the most important learning objective of the specific simulation is. Otherwise, it might be forgotten or overthrown by other impressions from the SP’s performance. Knowing the learning objective helps the SP assess the meaning of events and artefacts within the scenario. Not having oxygen would be irrelevant for some learning goals (e.g., bringing bad news) but relevant for others (e.g., making sure that necessary equipment is available). Knowing the learning objective allows SPs to assess whether the omission of devices and processes or changes in their use are relevant for the scenario or not and to get an idea about how to adjust their performance.

Another important subject in the meaning aspect is to clarify what the learners are supposed to experience, and how SPs can contribute to this experience.

- What are learners supposed to experience?

Simulation has the potential to be a full-body learning experience, and it offers a very different way of learning than reading a book or listening in an auditorium. This potential is unique and should be deliberately explored and maintained in scenario-based simulations, acknowledging, of course, that one cannot determine others’ experience.^4^ This means that learners will meet the acuteness of the emergency created by the SP if the learning objective is to bring the emergency kit in a transport scenario, and the learners forgot. It means that learners will be interrupted by the patient, if a learning objective is to involve the patient in decision making, and they do not address the patient.

### Narrative: the patient’s background and how the scenario is supposed to unfold

The narrative is about the patient’s background and how the scenario is supposed to unfold. The narrative is not about the patient as a person but about the planned ‘story line’ of the simulation. This is usually described briefly in the scenario script, but an SP remarked that not only do scenario scripts often lack detail in this regard, but one of the most challenging and confusing aspects of being an SP in a scenario is to know ‘where we are’ in the scenario. Even if the narrative is detailed in the scenario script, the timing of developments can be difficult to recognize and manage for SPs.

Since facilitators sometimes adjust some of the details in the narrative, it is important to let the SP know about these changes before the scenario begins or find a way of communicating it as the scenario unfolds. If the narrative is described in detail and the facilitator has no adjustments, it is also important to assure the SP that they can rely on the scenario script.

- What happened before the beginning of the scenario?

One of the frequently used and most important pieces of information for the SP and learners alike is what happened before the scenario takes place. Has the patient been in her bed for a long time, did she just arrive, or is she in and out of the hospital on a regular basis? A key question to ask here is: what brings the healthcare professionals and patient together right now (e.g., the patient called for help)?

- How might symptoms develop?

SPs need instructions about what are the relevant symptoms in this specific scenario and how the presentation of these symptoms change over time and in response to the interventions that learners would most likely apply. A facilitator could say, “You feel like *this*, which often looks or sounds like *this*. Learners might give you *this* medicine, which will have *this* effect on you. If they do, you will be fine after three minutes”.

- What is the expected course of the scenario?

For the SP to act in a relevant way, they need to know the expected course of the simulation and the expected actions by the learners. It might also be useful to discuss with the SP in advance what to do if learners treat symptoms in a different or wrong way and how the SP should respond to that.

Finally, it might be relevant for the SP to know when the simulation ends. Does it end as soon as the learners choose or complete a treatment, or does it continue until the patient has been informed? The duration of the scenario is usually predefined, so it is helpful for the SP to know how much is supposed to happen during the allocated minutes.

### Style: tone and tempo of the simulation

Style is about how the simulation should be carried out. It is not about *what* happens, but about *how* it happens – almost akin to genre: Are we in a children’s tale, a detective riddle, or an action movie? Style may be reflected in the level of detail in cues to learners, in the kind of actions that the script and the setting make available, and in the regulation of pace and urgency. The style aspect is rarely explicitly prescribed in scenario scripts, so both facilitators and SPs are relatively free to create this aspect of the simulation and thereby to adjust it to learners’ skills and signs of stress; we have seen that both facilitators and SPs do utilize this freedom. Style is closely tied to the meaning and narrative aspects of the simulation in the sense, that style determines how the meaning and narrative are to be conveyed. Style determines how the simulation should feel. Should a wrong decision by learners be met by a stressful deterioration of health or rather to more explicit cues to help the learners getting back on track?

- How - and how explicitly - should the ‘narrative’ and ‘meaning’ be presented to learners?

The facilitator often has a good idea of the learners’ level of experience, so they have a better basis for assessing the needs of the learners than the SP has. If the learners are new to simulation and slightly nervous, the facilitator can tell the SP to be very explicit in the words of choice. Instead of saying “it hurts”, for example, they could say “it *really* hurts from my chest moving towards my shoulder”. Even though the last sentence might not be a realistic sentence coming from a patient, it can help learners complete the scenario successfully and better understand what to look for in future scenarios. Only telling the learners that something hurts, may prompt them to post better questions or contemplate more tests.

- Which expressions, means and effects can be deployed?

Should the SP respond clearly or more evasively to questions? Should the SP actively display a wound or a concern, or should the SP let learners uncover it themselves? Should necessary - and perhaps unnecessary - remedies and utensils be located very visibly in the room or do learners have to ask for them?

- How quickly should the scenario develop?

How soon should cues come, and how severe should the problem seem to the learners? If the scenario script describes a narrative beginning with a patient suffering from a decreased level of consciousness going towards coma, should the simulating participant be mumbling incoherently for the first few minutes or pass out as soon as the learners enter the room?

- How much pressure should be put on the learners? What kinds of pressure?

If learners are more experienced, the facilitator can ask the SP to challenge learners by talking constantly and demanding attention or by saying things that may complicate decision making, for example, by sharing irrelevant information that could be interpreted as a different illness. They may also get sick from prescribed medicine, throwing up and confusing the learners with symptoms that aren’t directly related to the main illness.

In our experience when teaching the pre-roling framework, style is the aspect of the framework that is most difficult to separate analytically from the other aspects. This may be because style in many ways is the result of all the other elements in the simulation being combined. Nevertheless, it is relevant to relate to style as a separate aspect of the simulation for at least two reasons. First, thinking through style can be a way of checking the coherence of all the pre-roling aspects in the simulation. Second, style is the aspect that most facilitators do adjust and customize from session to session for their learners. These are adjustments that SPs need to be informed about.

### Character: characteristics of the simulated patient

The character is about the figure that the SP is enacting. It is about portraying a patient, a relative, or a colleague as truthfully and convincingly as possible. This is the aspect of an SP’s work that has received the most attention in the literature on SPs ^1,2,11,24^. In actual practice portrayal can be disturbed. It happens that a facilitator has a completely different image of a character, for example a 60-year-old woman, than the SP. If the facilitator tries to correct the SP’s portrayal of the character as the simulation unfolds (e.g. if both SP and facilitator work from behind a screen in an operator room) this can lead the SP to question every subsequent move and ultimately hesitate so much that the portrayal becomes stifled.

It is important to go through these questions together *before* the scenario, because once the scenario is running the character should be the responsibility of the SP. Since the character is primarily the responsibility of the SP, the SP should take the lead on this part of the pre-roling with the facilitator. The SP often have a certain person in mind and should share these characteristics with the facilitator in order to be on the same page. The facilitator then offers input and adjustments. In order for the SP to respond and improvise truthfully, it is important that the SP knows:

- Who is the person “behind” the patient? (Age, family, job, etc.)

How old is the person? Does the person have a family? What does the person do for a living?

- How does the person look, talk and smell? (dress, voice, dialect, choice of words, body language, etc.)

Does the person have a distinct elderly voice, does the person speak a particular regional dialect or sociolect, and how does the person carry him or herself? In this regard it might be important to reflect together on how to avoid caricature [26].

- How does the person (re)act? (small talk style, jokes, opinions and phobias)

The last question in the character aspect points to some traits that make up the person, but also supplies the raw material for filling in gaps in conversation. These are sentences that do not affect the medical diagnosis directly, such as “Do you think it might be dangerous?”. Another characteristic could be an opinion against pain-easing medicine, performing a phobia of needles, or possibly even lashing out at the learners.

Having covered the dramaturgical aspects of a simulation scenario, the facilitator and the SP are ready to perform. Distinct from the work of Shakespeare actors, however, the purpose of facilitators and SPs’ work is not only to perform but also to educate. For this reason, we have added health professional education to the framework. At this point we leave action theory to pragmatically respond to the needs expressed by our course leader colleagues at CAMES who design courses and scenarios, who oversee the running of the courses, and who sometimes facilitate simulations and debriefings. Some are also engaged in faculty development of external facilitators, but not in the training of SPs. In this section of the framework, we also loyally reflect an important portion of CDI’s ethnographic observations that does not fit directly into theories of performance or with the purpose of health professional education, but that is intimately linked to the practice of engaging SPs in scenario-based simulations, i.e. the well-being of SPs at work [10].

### Health professional education: the main purpose of simulation

In the process of developing the pre-roling framework we had questions and feedback from our course leader colleagues and SPs, who asked for a few more items to make the framework more complete for their needs. In pragmatic response to these requests we left theatre theory as an organizing principle of the framework. To cover the operational needs of our colleagues we added four items to consider when preparing together or individually for a simulation scenario: (1) the clinical picture, (2) simulation as a learning environment, and (3) responsibilities and distribution of tasks. The last item, (4) balancing fidelity, immersion, and care for the SP, grew from a recurring observation in CI’s data about SPs’ commitment to their task and the potential risk to their well-being that followed from that. Staying loyal to this observation we elaborate below.

These aspects are shown on the other side of the pocket card. The items here are not about how to act in front of specific learners in specific scenarios but about how SPs should be prepared if they do not yet know how, for example, delivery pains or asthma sounds or the effects of various medications. SPs can also use this side of the pocket card as a checklist for their questions to facilitators.

#### The clinical picture

If SPs are not patients or health care professionals themselves (yet) and therefore do not know how symptoms might evolve, they need to know about the specificities of symptoms more broadly - irrespective of the character suffering from them and the narrative in the scenario.

- What do specific symptoms look like? How does breathing sound?
- How do the most obvious choices of treatment affect symptoms? How quickly?

This information may be a lot to comprehend and remember the first few times, but by given the opportunity to ask these questions, the SP will quickly learn these medical facts.

#### Simulation as a learning environment

Sometimes the SPs’ experience can be useful to explore during debriefing.

- Should the experience of the SP be brought into the debriefing with learners?

The SP can remain in character when joining the debriefing or participate as her- or himself. This needs to be decided and communicated before the simulation begins so that the SP can take mental notes during the simulation. This is particularly relevant if the SP is not only expected to recall their experience in the scenario but also to verbalize it and perhaps even evaluate learners’ performance.

- How can learners be helped through the scenario?

Sometimes, learners move off-track and away from the learning goals of the scenario. Here it is useful for the facilitator and SP to share beforehand and agree on how to apply various “scenario life savers” to get the learners back on track [9].

#### Responsibilities and distribution of tasks

SPs’ tasks vary greatly depending on whether they enact their character as a voice from behind a screen or in body in the same space as learners. Novice SPs may be uncertain about their responsibilities particularly when they are operators behind the pane. Taking notes on participants’ performance is by default the responsibility of the facilitator. At CAMES giving voice to the manikin is usually the responsibility of the SP. Tasks, such as controlling the vital parameters, being the voice of God, handing over test results, and answering the phone from the simulation room, however, can be distributed between the facilitator and SP. Often, many of these tasks need to be carried out more or less simultaneously, so it may be useful to agree before the scenario, who does what. New facilitators, in particular, may sometimes assume that their main responsibility is to take notes of the participant’s performance, so they may leave many tasks for the SP to handle. For this reason, it is useful to clarify before the simulation:

- Where should the facilitator and the SP direct their attention during the simulation?

and.

- What signals are to be used during the scenario?

If the SP is in the room with the learners and the facilitator, verbal communication between the SP and the facilitator could disturb the “reality” of the scenario. In this case, it is useful to agree on signs or signals to apply during the scenario. This can be communicated with touch (pulling at a toe, if the SP is in a bed) or sounds (cough, code words).

#### Balancing fidelity, immersion, and care for the SP

SPs often take their responsibility for the quality of simulations very seriously, and they take pride in performing well [27]. This mix of immersion and taking responsibility for the level of fidelity and the learners’ experience sometimes leads them to inadvertently compromise their physical and mental well-being [25]. Surprisingly, SPs seem to downplay this as a risk, and they perceive the collapse of the illusion in the simulation as a greater risk. However, simulation centres are responsible for preventing harm to SPs. Therefore, we have incorporated into our framework a reminder to SPs and facilitators to pay attention to the ways that a scenario, a character, or a situation could become uncomfortable, and to agree on signals to apply to redirect or stop the unfolding of events. This is crucial since the SPs might risk their own well-being to minimize the need for fiction cues to keep the simulation on track [22]. Besides, de-roling may be relevant. In simulation de-roling has been addressed in relation to learners [28], but curiously and significantly not in the same detail in relation to SPs [29, 30].

We have summarized the five aspects, their significance, relevant questions to ask for each aspect, as well as the gains of addressing the questions and risks of not addressing them in Tables 1, 2, 3, 4 and 5. Based on CDIs observation of misunderstandings and dissonances during fieldwork, the tables offer a practical overview of questions and how they are justified in the framework.

**Table 1:** Meaning

Significance	Questions	Reward	Risk
Attuning the main target for the simulation to make sure that both the SP and facilitator emphasize the required actions to train the learners for the learning objective.	What is the most important learning objective?	The meaning should facilitate that the SP and facilitator are working towards the same endpoint of the simulation	When the meaning is not aligned, the SP might improvise in a direction, that undermines the main learning objective of the simulation
		The SP can comfortably improvise during the simulation, since it is clear whether the SP is moving in the wanted direction	When the SP is uninformed of the meaning, the SP might second guess every step within the scenario, which will harm the fidelity of the simulation
Articulating what sensations, the SPs should aim to make the learners experience	What are learners supposed to experience?	Whenever the learners are acting against the learning objective, the SP will know how to emphasize the impact of these actions.	Without attuning this step, it will be unclear how the SP should react to the actions of the learners.

**Table 2:** Narrative

Significance	Questions	Reward	Risk
The narrative informs the SP of the preceding events, leading up to the scenario.	What happened before the beginning of the scenario?	The SP can utilize the background information to create a more coherent scenario, improving the fidelity.	The SP might hesitate answering basic questions, if the background story is not clear.
The narrative relates to how the scenario should and might develop	How might symptoms of the develop?	Gives the SP an itinerary for the scenario, which makes it able to improvise in concord with the wanted development	Without knowing the wanted direction for the scenario, the SP might improvise in the wrong direction.
	What is the expected course of the scenario?	Gives the SP the necessary information to react to the operations of the learners. The SP will know when to get worse or better.	The SP won’t be able to react correctly to the learners’ actions, which might lower the fidelity of the simulation.

**Table 3:** Style

Significance	Questions	Reward	Risk
The style determines how clearly the meaning should be delivered and how much help the learners should get.	How - and how explicitly - should the narrative and meaning be presented?	The SP can challenge the learners the right amount and make sure that they reach the learning object of the scenario	The SP might make the meaning so unclear, that the learners start going in the wrong direction or so obvious that they figure it out right away
The style determines how fast the narrative should develop and how intensely the symptoms should be delivered.	Which expressions, means and effects can be deployed?	The SP is informed of explicit ways to help or challenge the SP’s. For example, by explaining the symptoms very explicitly if the learners are struggling or putting emphasize on misleading symptoms if the learners are doing too well.	If it is unclear to the SP, how to make the symptoms more or less evident, they are unable to help the learners during the scenario.
	How quickly should the scenario develop?	By knowing the wanted pace of development, the SP can make sure, that the scenario unfolds appropriately.	The SP might move too fast, making the learners panic or too slow, making the learners bored.
The style determines how much space the character should take up and how cooperative the character should be.	How much pressure should be put on the learners? What kinds of pressure?	The SP can adjust their performance in concord with the learners’ level of experience and make sure that they progress in the scenario	The SP might put too much pressure on the learners, making them unable to move forward in the scenario.

**Table 4:** Character

Significance	Questions	Reward	Risk
The character is defining the characteristics of the patient that are not directly linked to the diagnosis but are important for creating a realistic scenario. It determines who the person behind the patient is, how this person responds and what the person wants.	Who is the person “behind” the patient? (Age, family, job, etc.)	Being able to comfortably answer questions and engage in “natural“ interactions.	
	How does person look, talk and smell? (voice, dialect, choice of words, body language etc.)	Helping to find one’s feet in the portrayal of the character, enhancing the joy of acting.	Experience of pressure, if one is not comfortable with acting and portraying a role. Memory and cognitive load that needs to be mastered.
	How does the person (re)act? (small talk style, jokes, opinions and phobias)		

**Table 5:** Health professional education

Significance	Questions	Reward	Risk
The clinical picture: The professional aspect of the simulation and should inform the SP about the medical aspects of the diagnosis since the SP rarely know this by heart.	What do specific symptoms look like? How does breathing sound?	Ensuring plausibility and consistency in the scenario. Easy of mind for the SP, being as prepared as possible.	Many things to remember, pressure from the responsibility to not getting this wrong.
	How do the most obvious treatments affect symptoms? How quickly?		
Simulation as a learning environment: Extent to which SP’s experience during the simulation should feed into the debriefing and what aspects of the learning objectives the SP should provide feedback on.	Should the experience of the SP as patient feature in the debriefing of learners?	The SP can bring in feedback from different roles into the debriefing.	SPs need to be able juggle different aspects for their experiences and feedback from the “right” perspective to not get confused and to not confuse.
	How can learners get help? How much help?		
Responsibilities and distribution of tasks: Distribution of the responsibilities between the SP and facilitator, making the tasks evident for the SP.	How should the faciliator and SP divide their attention during the simulation?	Coordinated actions for a consistent and plausible scenario. As well as clear ground rules for possible scenario life savers.	Need to attune into each others’ expectations, memory burden.
Agreement on signals for adjusting the simulation en route.	What signals can be used during the scenario?		
*Balancing fidelity*,* immersion and care for the SP*	How does the SP feel about the role and its practical, physical and emotional implications?	Risks can be anticipated and mitigated (e.g., limits on emotional intensity, touch, aggressive language, physical tasks), and de-roling/support can be planned—protecting SP well-being while maintaining sufficient fidelity.	The SP may downplay strain and push through to protect the illusion, increasing risk of physical/mental harm, emotional spillover after the session, and eventual reduced performance or withdrawal.

## Considerations and practical tips for implementation

The pre-roling framework is intended to be applied by SPs and facilitators before a simulation. Preferably this is done together, but it can also be done individually as preparation for the most important questions and instructions to each other. It might be helpful to experiment with different formats, e.g., the pocket cards (additional file 1) or a table format, like the pre-roling sheet (additional file 2), where the first works as a checklist to go over individually and together, and the second invites to written individual preparation that can be shared later.

A central consideration is the time that should be built into simulation programmes to pre-role for both SPs and facilitators. It is important to emphasize that not all items in all aspects of the framework need to be addressed before all scenarios. The specific context determines which aspects are most important to cover, but as a minimum meaning should always be made explicit. When the same SP and facilitator perform the same scenario together for the second time, a shortened version of pre-roling might suffice. We could call it re-roling, where a simple check: “Same meaning, narrative, and style as last time?” might suffice. Once a new SP or facilitator has gained some experience or once a new scenario has become well-known to SPs and facilitators, the main use of the pre-roling framework might be as a systematic but selective checklist for ad hoc adjustments rather than as a comprehensive point-by-point agenda for a meeting. It might still be useful to mention every aspect to minimize the risk that assumptions differ too much with respect to meaning, narrative, style, or character and how to fine-tune collaboration between SP and facilitator. It may happen that conflict between interpretations occur. Rather than prescribing what aspect is most important we suggest facilitator and SP find time to discuss how the meaning of the scenario, insights into learners’ needs, and forms of fidelity [19] are best balanced.

The pre-roling framework has grown out of a particular set of observations in a particular organizational setting at CAMES in Copenhagen. Whether the framework is relevant in other settings may be influenced by the frequency with which new SPs, new facilitators, and new scenarios are introduced. If a setting for simulations is very stable or standardized in terms of people or scenarios, the pre-roling framework may be less useful. Another central consideration is the background and experience of SPs as patients and as actors. If SPs are people with lived experience as patients, if they are older [16], or if they are professional actors, the aspects in the framework can be the same, but emphases and process might need adjustment.

Summing up, we will draw attention to six characteristics of the framework that may make it relevant beyond the existing guidance for simulation designers, facilitators and SPs.

First, the pre-roling framework encompasses attention to both the dramaturgical, the health professional educational, and the collaborative aspects of simulation. Much published guidance focuses mainly on one or two of these aspects. Second, the framework has been developed and tested in a simulation center to make it as immediately operational as possible for both experienced and less experienced SPs and facilitators. Whereas knowledge of and experience with scenario-based simulation is useful, no particular understanding of acting theory or simulation theory is needed to apply the framework. It should be easily applied in most scenario-based simulation settings. Third, most guidance is offered to designers and facilitators whereas the pre-roling framework can be applied by SPs and facilitators in a symmetric way. Both SPs and facilitators can pick it up and start imagining a scenario script coming alive before meeting the other to clarify what is needed and expected from each other. Fourth, the pre-roling framework can help facilitators and SPs make preferences and adjustments explicit - particularly with respect to ‘style’, which is an aspect of performance that is not often addressed explicitly and where facilitators typically have specific preferences. Fifth, the framework enables tighter alignment between SP and facilitator through systematic preparation, but once the scenario is rolling the framework proposes that meaning, narrative and style are the main responsibility of the facilitator, whereas character is the main responsibility of the SP. This clear division of responsibilities may make it easier for both facilitators and SPs to focus their efforts although success still depends on continuous attunement between the facilitator and the SP. Sixth, the framework explicitly includes care for the SP. Attention to SPs’ protection and experience during the scenario is as central as attention to learners’ protection and experience.

Going through the pre-roling framework is no guarantee that misunderstandings and dissonance will be avoided completely, but it enhances the likelihood that SPs and facilitators are aligned regarding pivotal aspects of the simulation before it is time for action. We welcome local adaptations of the pre-roling framework with language and items that may be more familiar and relevant but recommend that the analytical distinction between aspects will be maintained.

## Conclusion

We propose a pre-roling framework based on anthropological action theory for the SP and the facilitator to prepare systematically together for scenario-based simulations. The pre-roling framework embraces both dramaturgical, health professional educational and collaborative aspects of the simulations and could help enable better attunement between the facilitator and the SP before and during the simulation. The pre-roling framework reduces guesswork and the risk of dissonance between SP and facilitator by enabling a clarification about the meaning, narrative, style, character and health professional educational aspects of the simulation scenario. Better preparation increases the likelihood of a useful learning experience for learners, which is also safer for the SP. In this way, the pre-roling framework not only prepares SPs for better acting and teaching, but also enables a safer working experience.

The pre-roling framework is flexible and adaptable to various simulation settings. Our aim is to systematize preparations in an operational manner to both enable relevant improvisations, thereby improving simulation fidelities and learning potentials, and to minimize the risk that SPs embodying patients, relatives, caretakers, and confederates experience unaddressed distress.

## Supplementary Information

Additional file 1: Pre-roling pocket card.

Additional file 2: Pre-roling sheet.

## Data Availability

The datasets generated and/or analysed during the current study are not publicly available due to anonymity and confidentiality promised to study participants. Datasets may be made available from the corresponding author on reasonable request.

## Footnotes

1 ‘Pre-roling’ is inspired by the term ‘de-roling’, which is the application of a range of physical and mental techniques, such as removing make-up and costume, singing a song, or shaking one’s body to shed intense roles in theatre and drama therapy [31].

2 Building on the term ‘simulated participant’ as a cover term for people enacting roles as patients, relatives, colleagues, or other healthcare professionals [30], we apply the term ‘simulating participant’ to emphasize our attention to the person who enacts a role in a scenario.

3 In this paper the term ’learners’ refer to HPE-students or the learning participants in simulations.

4 Interestingly, when presenting the pre-roling framework in workshops and conferences participants often stumble over the term ‘experience’. Why not ‘learn’? Why not just omit the question? We keep the question in the pre-roling framework, and we keep the broad term ‘experience’ because this is exactly one of the strengths of simulation as a learning modality.
