# Supplementary material for: Pre-roling: an operational framework for facilitators and simulating participants (SPs) to prepare for both acting and educating safely
Source: Adv Simul (Lond). 2026 Feb 23;11:27. doi: 10.1186/s41077-026-00419-w (PMC13040827; doi:10.1186/s41077-026-00419-w)
Supplement: Supplementary file 1 — Additional file 1: Pre-roling pocket card. [file 41077_2026_419_MOESM1_ESM.pdf]

PRE-ROLING

A framework for faciltators and simulating participants to prepare systematically for scenario-based simulations

Copenhagen Academy for Medical Education and Simulation (CAMES)

CHARACTER: Characteristics of the simulated patient

- Who is the person “behind” the patient? (Age, family, job, etc.)
- How does person look, talk and smell? (clothes, voice, dialect, choice of words, body language, etc.)
- How does the person (re)act? (small talk style, jokes, opinions and phobias)

MEANING: Purpose of the simulation

- Most important learning objective?
- What are learners supposed to experience?

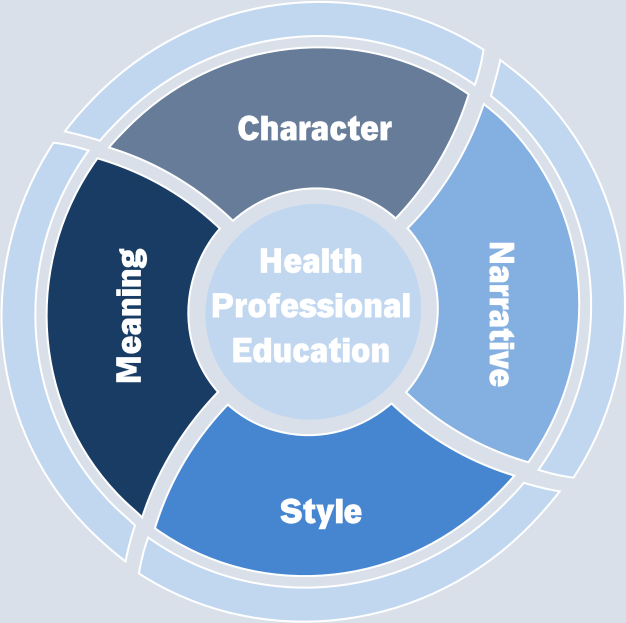

NARRATIVE: The patient’s background and how the scenario is supposed to unfold

- What happened before the beginning of the scenario?
- How might symptoms develop?
- What is the expected course of the scenario?

STYLE: How should the simulation be carried out?

- How - and how explicitly - should the Narrative and Meaning be presented?
- Which expressions, means and effects can be deployed?
- How quickly should the scenario develop?
- How much pressure should be put on the participants? What kinds of pressure?

PRE-ROLING

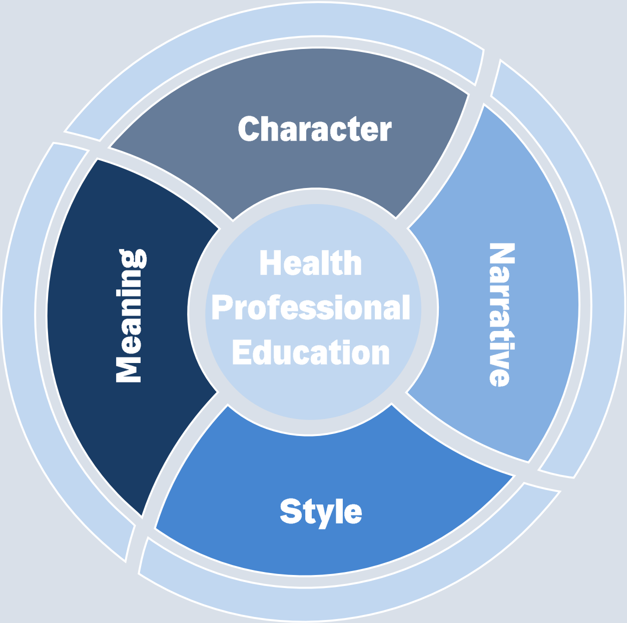

EDUCATION: The main purpose of the simulation

The clinical picture

- What do specific symptoms look like? How does breathing sound?
- How do the most obvious choices of treatment affect symptoms? How quickly?

Simulation as a learning environment

- Should the SP's experience figure in the debriefing of learners?
- How can learners get help? How much help?

Responsibilities and distribution of tasks

- Where should the facilitator and SP direct their attention during the simulation?
- What signals are to be used during the scenario?

Balancing fidelity, immersion and care for the SP

- *How does the SP feel about the role and its practical, physical and emotional implications?*

Copenhagen Academy for Medical Education and Simulation (CAMES)
