# Supplementary material for: Pre-roling: an operational framework for facilitators and simulating participants (SPs) to prepare for both acting and educating safely
Source: Adv Simul (Lond). 2026 Feb 23;11:27. doi: 10.1186/s41077-026-00419-w (PMC13040827; doi:10.1186/s41077-026-00419-w)
Supplement: Supplementary file 2 — Additional file 2: Pre-roling sheet. [file 41077_2026_419_MOESM2_ESM.pdf]

PRE-ROLING

MEANING: Purpose of the simulation
Most important learning objective?
What are learners supposed to experience?

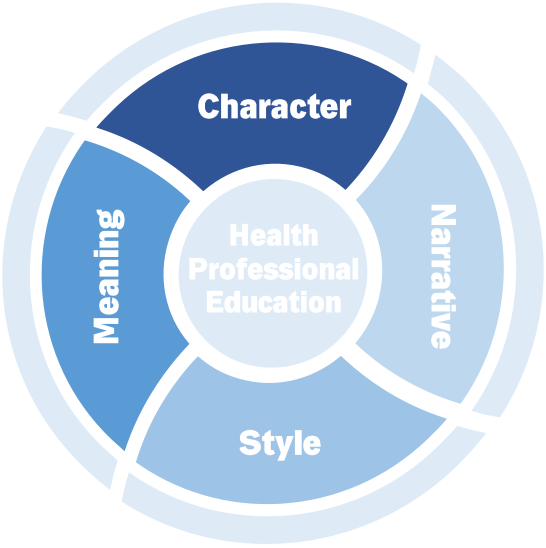

NARRATIVE: The patient's background and how the scenario is supposed to unfold.	
What happened before the beginning of the scenario?	
How might symptoms develop?	
What is the expected course of the scenario?	

CHARACTER: Characteristics of the simulated patient	
Who is the person "behind" the patient? (Age, family, job, etc.)	
How does person look, talk and smell? (clothes, voice, dialect, choice of words, body language, etc.)	
How does the person (re)act? (emotional state, small talk style, jokes, opinions and phobias)	

**<u>STYLE</u>: How should the simulation be carried out?**	
How - and how explicitly - should the narrative and meaning be presented?	
Which expressions, means and effects can be deployed?	
How quickly should the scenario develop?	
How much pressure should be put on the learners? What kinds of pressure?	

**<u>EDUCATION</u>: The main purpose of the simulation**	
**The clinical picture:**<br>• What do specific symptoms look like? How does breathing sound?<br>• How do the most obvious choices of treatment affect symptoms? How quickly?	
**Simulation as a learning environment:**<br>• Should the SP's experience figure in the debriefing of learners? How?<br>• How can learners get help? How much help?	
**Responsibilities and distribution of tasks:**<br>• How should facilitator and SP divide their attention during the simulation?<br>• What signals are to be used during the scenario?	
Balancing fidelity, immersion and care for the SP:<br>• *How does the SP feel about the role and its practical, physical and emotional implications?*	

<u>Other notes:</u>
